# Prion Seeding Activity in Plant Tissues Detected by RT-QuIC

**DOI:** 10.3390/pathogens13060452

**Published:** 2024-05-26

**Authors:** Kate Burgener, Stuart Siegfried Lichtenberg, Daniel P. Walsh, Heather N. Inzalaco, Aaron Lomax, Joel A. Pedersen

**Affiliations:** 1 Molecular and Environmental Toxicology Program, University of Wisconsin-Madison, Madison, WI 53706, USA; kburgen1@jhu.edu (K.B.);; 2 Department of Environmental Health and Engineering, Johns Hopkins University, Baltimore, MD 21218, USA; 3 Department of Veterinary and Biomedical Sciences, University of Minnesota, St. Paul, MN 55108, USA; 4 Minnesota Center for Prion Research and Outreach, University of Minnesota, St. Paul, MN 55108, USA; 5 U.S. Geological Survey, Montana Cooperative Wildlife Research Unit, University of Montana, Missoula, MT 59812, USA; 6 Wisconsin Cooperative Wildlife Research Unit, Department of Forest and Wildlife Ecology, University of Wisconsin-Madison, Madison, WI 53706, USA; 7 Department of Soil Science, University of Wisconsin-Madison, Madison, WI 53706, USA; alomax@varizymes.com; 8 Varizymes, Middleton, WI 53562, USA

**Keywords:** prions, plants, chronic wasting disease, real-time quaking induced conversion, seeded amplification, environmental transmission

## Abstract

Prion diseases such as scrapie, bovine spongiform encephalopathy (BSE), and chronic wasting disease (CWD) affect domesticated and wild herbivorous mammals. Animals afflicted with CWD, the transmissible spongiform encephalopathy of cervids (deer, elk, and moose), shed prions into the environment, where they may persist and remain infectious for years. These environmental prions may remain in soil, be transported in surface waters, or assimilated into plants. Environmental sampling is an emerging area of TSE research and can provide more information about prion fate and transport once shed by infected animals. In this study, we have developed the first published method for the extraction and detection of prions in plant tissue using the real-time quaking-induced conversion (RT-QuIC) assay. Incubation with a zwitterionic surfactant followed by precipitation with sodium phosphotungstate concentrates the prions within samples and allows for sensitive detection of prion seeding activity. Using this protocol, we demonstrate that prions can be detected within plant tissues and on plant surfaces using the RT-QuIC assay.

## 1. Introduction

Transmissible spongiform encephalopathies (TSEs) are fatal neurodegenerative disorders that affect mammals. The causative agents of TSEs are prions, misfolded proteins, which can catalyze the misfolding of normal prion proteins and are recalcitrant to degradation [1]. The detection of prions has historically been a complicated and challenging task. Antibody-based detection assays (ELISA, western blot) possess limits of detection far above infectious doses, limiting their effectiveness to high-titer samples [2]. Animal bioassays are far more sensitive, possessing a limit of detection that is by definition one infectious dose, and are ever more useful with the advent of transgenic methods and gene editing technologies [3,4]. However, due to the remarkably slow progression of TSEs, bioassays are expensive and time-consuming endeavors. Ultra-sensitive protein amplification assays, first realized as protein misfolding cyclic amplification (PMCA) [5,6] and later expanded to real-time quaking-induced conversion (RT-QuIC) [7,8], allow for the detection of prions with sensitivity that approaches or exceeds animal bioassay—which is the gold standard for assessing a sample for the presence of infectious prions—while providing interpretable data in a matter of days. The RT-QuIC assay is an amyloid detection assay that uses the ability of prions to template the conversion of normally folded prion protein to misfolded conformers [7]. In RT-QuIC, a sample is incubated in a mixture of recombinant prion protein while alternating cycles of shaking and incubation occur. The output is a continuous measure of thioflavin T (ThT) fluorescence, a dye that binds to and is stabilized by amyloid fibrils [9]. Binding of thioflavin T to amyloid fibrils results in increased fluorescence intensity and a bathochromic shift in the maximum emission spectra, which can be observed as a function of time in RT-QuIC [10]. RT-QuIC is highly sensitive and specific for prions and can produce results within hours [11]. The real-time measurement allows for a semi-quantitative analysis of amyloid formation for each sample. Commonly reported results are time-to-threshold measurements, maximum detectable dilution, and the max point ratio [12]. Rapid time-to-threshold and seeding activity at very dilute concentrations correspond to high concentrations of seeding material. RT-QuIC has a sensitivity comparable to animal bioassays while eliminating the need for transgenic animal colonies required for bioassay and PMCA [2], with the pitfall that information related to strain differentiation is not detectable using RT-QuIC alone and the product cannot be subsequently tested for infectivity using bioassay.

Certain prion diseases display pronounced lymphtropism and associated prion-shedding behaviors in peripheral tissues and fluids [13,14,15,16,17,18]. This seems to be particularly pronounced with chronic wasting disease (CWD), the TSE of cervids (deer, elk, and moose) [19,20,21,22]. Combined with the significant deposition from decomposing CWD-positive (CWD+) carcasses [23], this shedding behavior results in significant levels of environmental prion contamination. The full extent of the consequences of environmental prion deposition is not fully known, but it is widely accepted that environmental prion deposition likely leads to disease transmission [24] and could reasonably play a major role in the ultimate outcomes of CWD dynamics [25,26]. In addition to soil, water, and other surfaces, plants are being studied for their potential as a transmission source and prion reservoir in the environment. Plants are in constant contact with soil and water and are consumed by cervids, bovids, and ovines. When grown in environments where prions are present, plants can uptake and translocate prions to roots, stems, and leaves. Most alarmingly, prions associated with plants retain infectivity [27,28].

Methods for analyzing samples with RT-QuIC have been established for animal tissues [15,16,29,30,31,32,33]. Though radically different in physiology, plant and animal tissues possess most of the same biomolecules, with exceptions mainly found in the constituents of the plant cell wall [34]. In addition, samples with complex matrices such as soil, feces, and plants often have low concentrations of prions and constituents, which may lead to interference with the assay [35,36,37,38]. Due to the extreme heterogeneity of these samples, the precise effects of the assay and which molecules or surfaces are causing such effects may not be readily identified. These interferences could take the form of false negatives, false positives, or inconclusive results [31,37,38]. Analyzing complex and low-abundance samples often requires specialized extraction methods such as iron-oxide magnetic bead adsorption and phosphotungstic acid precipitation to produce clear results [16,39,40,41,42]. Plant tissue is a heterogeneous conglomerate of cellulose, proteins, and sugars. These macromolecules may be obstructive or photoactive, which can impede RT-QuIC fluorescent measurements [43,44,45,46]. Initial experiments revealed that the presence of plant homogenates substantially inhibits RT-QuIC reactions (Supplementary Figure S1). Therefore, the development of methods that reduce or remove interfering compounds prior to analysis is essential. To gain a clear understanding of the role plants play in the environmental transmission of prion diseases, a method for sample preparation that is compatible with RT-QuIC is necessary. In the present study, we aimed to create a method of prion extraction that concentrates PrP^TSE^ from plant samples and removes inhibitory plant matter. *Arabidopsis thaliana* (mouseear cress), *Brachypodium distachyon* (purple false brome)*,* and *Zea mays* (maize) were used for proof-of-concept analyses. In addition, we demonstrate the utility of this method using plant samples that were previously shown to have assimilated infectious prions [28].

## 2. Materials and Methods

### 2.1. Chemicals and Reagents

All buffers and solutions were prepared with 18 MΩ sterile distilled H_2_O, unless otherwise noted. Myristyl sulfobetaine (MSB) buffer was prepared using 0.6 mM myristyl sulfobetaine (Sigma-Aldrich, T7763, St. Louis, MO, USA), 75.4 mM dibasic sodium phosphate (Sigma-Aldrich, S9763, St. Louis, MO, USA), and 24.6 mM monobasic sodium phosphate (Sigma-Aldrich, S0751, St. Louis, MO, USA). The final pH was adjusted to 7.4. Sodium phosphotungstate (NaPTA) solution was made by dissolving crystalline sodium phosphotungstate to a 6.8% mass/volume concentration. The final pH was adjusted to 7.1 with 10 M sodium hydroxide. The RT-QuIC sample buffer was prepared as previously described [47], using 990 µL of 0.1% sodium dodecyl sulfate (Fisher Scientific, BP166, Waltham, MA, USA) in 1× phosphate buffered saline and 10 µL of N-2 MAX Media Supplement (R&D Systems, AR009, Minneapolis, MN, USA). Similarly, RT-QuIC reaction buffer was prepared as 1× PBS, 170 mM sodium iodide (LabChem, LC246451, Zelienople, PA, USA), 1 mM ethylenediaminetetraacetic acid (IBI Scientific, IB70182, Dubuque, IA, USA), 0.1 mM Thioflavin T (Sigma-Aldrich, SHBL4963, St. Louis, MO, USA), and 0.1 mg/mL truncated recombinant hamster prion protein (residues 90–231), synthesized as previously described [47,48].

### 2.2. Sample Sources

CWD+ brain samples were sourced from deceased, radio-collared white-tailed deer that were a part of a Wisconsin Department of Natural Resources (WDNR) CWD monitoring project. Positive status was confirmed by immunohistochemical analysis and RT-QuIC. CWD-negative brain samples were provided by the Texas Parks and Wildlife Department from a population of white-tailed deer culled for population control in Texas. The source population is geographically isolated from any known CWD cases, and the negative samples have previously been confirmed to be negative for prion seeding activity by both RT-QuIC and protein folding misfolding cyclic amplification.

Plant samples were sourced from the U.S. Geological Survey National Wildlife Health Center, where they were part of a prion-plant project from 2008 to 2014 [28]. *Sativa medicago* (alfalfa) and *Hordeum vulgare* (barley) were hydroponically grown for four weeks in water spiked with sequentially digested whole brain homogenate from clinically terminal animals infected with the RML strain of mouse-adapted scrapie. Following exposure, the peripheral leaves and stems were harvested and stored at −80 °C.

### 2.3. Spiking Experiments

For spiking experiments, whole plants were harvested, and all aerial plant tissue (leaves, stems, and nodes) was flash frozen with liquid nitrogen, powdered, and kept in a 1.5 mL polypropylene microcentrifuge tube at −80. For every 0.1 g of powdered plant tissue, 100 µL of 10^−2^ brain homogenate (BH) dilutions in PBS were added. Following the addition of BH, samples were treated with the prion extraction protocol described below, except for the first step of freezing and powdering the sample.

### 2.4. Rinsing and Washing Experiments

*Elymus repens* (couch grass) leaves were cut into 0.1 g segments. A n amount of 100 µL of brain homogenate (BH) diluted to 10^−2^ in water was deposited onto the surface of each leaf segment, and the liquid was allowed to evaporate for 16 h at room temperature. Negative controls were constructed similarly, with H_2_O only and CWD-negative BH on *E. repens* leaves. CWD+ BH was used for the three experimental conditions: control, soak, and rinse. When the liquid had completely evaporated, the leaves in the soaking group were submerged in 6 mL of water for 10 s. The rinsing group leaves were rinsed with six milliliters of water for 10 s at a volumetric flow rate of 36 mL/min. The control group did not receive any additional treatment. All samples were then extracted using the prion extraction method described below and analyzed by RT-QuIC.

### 2.5. Prion Extraction

Plant tissues were frozen with liquid nitrogen and finely ground with a mortar and pestle. Plant powder (0.1 g) was added to 500 µL of MSB solution and vortexed vigorously for 15 min. Samples were sonicated in a cup-horn sonicator (Qsonica Q700-110, Newtown, CT, USA) for 2 min at an amplitude of 36 and subsequently vortexed vigorously for an additional 15 min. Samples were then centrifuged at 16,000× *g* for 30 min at room temperature. Following centrifugation, 300 µL of supernatant were removed and set aside. An amount of 500 µL of MSB solution was added to the remaining plant extract pellet, and samples were vortexed vigorously for 30 min, then centrifuged at 16,000× *g* for 30 min at room temperature. A 400 µL aliquot of supernatant was taken from the extract and combined with the 300 µL from the previous supernatant. Then, an equal volume of 6.8% NaPTA (pH 7.1) was added to each supernatant, gently mixed, and incubated at 20 °C overnight (at least 16 h). Following incubation, supernatants were centrifuged at 10,000× *g* for 30 min at 4 °C. Supernatant was carefully removed while avoiding the small pellet that formed. Gently, 100 µL of diH_2_O and 100 µL of 6.8% NaPTA were added to each sample pellet. Samples were then centrifuged again at 10,000× *g* for 30 min at 4 °C. This wash process was repeated one additional time. After removing the second wash supernatant, the pellets were resuspended in 180 µL RT-QuIC sample buffer. Samples were stored at 20 °C prior to analysis with RT-QuIC.

### 2.6. RT-QuIC

For each technical replicate, 2 µL of sample in RT-QuIC sample buffer was added to 98 µL of RT-QuIC reaction buffer in individual wells of a black 96-well clear bottom optical plate (Thermo Fisher Scientific, 265301, Waltham, MA, USA). The plates were sealed and incubated for 48 h at 50 °C in a FLUOstar Omega plate reader (BMG Labtech GmBH, Cary, NC, USA) with 60 s double orbital shaking at 700 rpm followed by 60 s of rest. Fluorescent measurements were taken every 15 min with a 448 nm excitation filter and a 482 nm emission filter, each with a 10 nm bandwidth. All samples were analyzed using eight technical replicates and were considered positive if four or more replicates exceeded a threshold of 10 times the standard deviation of the baseline fluorescence (the average relative fluorescence of cycles 3–13) before 48 h.

### 2.7. Statistics

Direct comparisons between two treatments were performed with two-sided *t*-tests, and analyses of three or more treatments were accomplished with one-way ANOVA. Statistic results were considered significant at *p* < 0.05. All statistics were performed with MATLAB and Statistics Toolbox version 9.9, release 2020b (The Math Works Inc., Natick, MA, USA).

## 3. Results

### 3.1. Extraction Optimization

Our extraction method first mechanically disrupts plant tissue to release any entrained prions within tissue and to allow solution contact with as much tissue surface area as possible. Myrisrtyl sulfobetaine is an organosulfonic acid. The RT-QuIC assay is extraordinarily sensitive to surfactant concentration [31]. Notably, increased concentrations of sodium dodecyl sulfate slow the kinetics of RT-QuIC but also stimulate spontaneous seeding in unseeded reactions using certain substrates. Prior studies have shown that myristyl sulfobetaine does not stimulate the spontaneous fibrillation of prion protein, making it suitable for use as an extractant for sample preparation prior to the RT-QuIC assay [49]. Repeated physical agitation combined with the MSB solution is thought to separate the PrP^TSE^ from plant macromolecules, as the proteins are soluble in the MSB solution and light enough to remain in the supernatant following intense centrifugation. Once most of the plant tissue debris is removed, a NaPTA precipitation can be performed. The addition of NaPTA reduces the solubility of the remaining proteins and PrP^TSE^. After incubation, the PrP^TSE^ can be separated from the supernatant by centrifugation. This pellet can be resuspended with RT-QuIC sample buffer and will remain stable until it can be analyzed by the assay.

Different extraction parameters were tested on the NaPTA precipitation step of the protocol to determine if there are any optimal conditions that improve the performance of the extraction (Supplementary Figure S2). Three different temperature conditions (4, 25, and 37 °C) and two precipitation times (1 and 16 h) were performed. One-way ANOVA tests were performed on the results from both precipitation time experiments. There was a significant difference in the mean time-to-threshold for the three temperatures in the 1-h precipitation (F(2,21) = 4.03, *p* = 0.033); however, there were no statistical differences between the mean time-to-threshold of the temperature for the 16 h precipitation (F(2,45) = 1.13, *p* = 0.333). Two-sided t-tests were performed comparing the same temperatures for the 1- and 16-h precipitation protocols. At 25 °C, there was a significant difference (t(22) = −2.414, *p* = 0.025) in the mean time-to-thresholds between the 1-h NaPTA precipitation (µ = 11.063, σ = 2.026) and the 16-h precipitation (µ = 13.453, σ = 2.400). There was also a significant difference (t(22) = −2.254, *p* = 0.035) between the 1-h (µ = 11.844, σ = 1.488) and 16-h precipitation (µ = 14.250, σ = 2.808) at 37 °C. At 4 °C, there were no significant differences (t(22) = 0.589, *p* = 0.562) between the 1-h (µ = 13.469, σ = 1.634) and the 16-h precipitations (µ = 12.844, σ = 2.748). Given that an extended time does not negatively impact results and the entire extraction and RT-QuIC preparation cannot be performed in a single day, we chose to incubate our samples for 16 h at 4 °C.

### 3.2. Spiking Experiments

To determine if interferences from plant tissue coextractants are produced by our extraction protocol, we spiked plant tissue homogenates with prion-positive brain homogenate. We chose to use a high concentration of prion-bearing material to ensure that a detectable signal was generated in the course of the assay and to ensure any interferences could be reasonably quantitated. The model plants, *A. thaliana* and *B. distachyon,* were chosen because of their widespread use in research and ease of growth. *Z. mays* (corn plant) was used because of its prevalence in agriculture and feedstock. Following the method outlined above, spiked plant extracts were analyzed for seeding activity with RT-QuIC.

**Figure 1:**
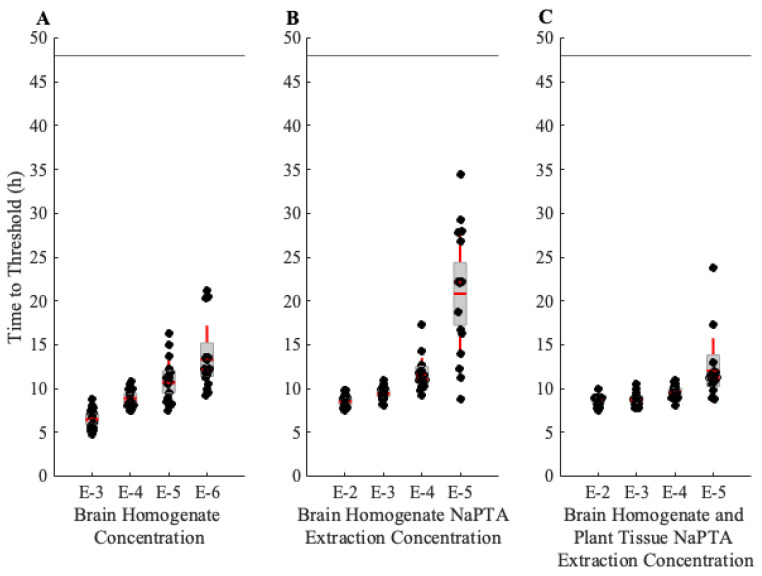
Real-time quaking-induced conversion (RT-QuIC) analysis of the prion extraction method efficiency. Box plots indicate the median time-to-threshold with a dashed horizontal red line, the mean with a solid horizontal red line, the second and third quartiles with the box, and the first and fourth quartiles with the whiskers. The horizontal line at 48 h indicates the end time of the assay. Each condition was performed with 16 technical replicates. (**A**) Threshold results from brain homogenate (BH) run at dilutions of 10^−3^ to 10^−6^. (**B**) Time-to-threshold results for BH subjected to the prion extraction method at dilutions of 10^−2^ to 10^−5^. (**C**) Threshold measurements for BH-spiked plant tissue (*Brachypodium distachyon*) subjected to the prion extraction method at dilutions of 10^−2^ to 10^−5^.

The positive control brain homogenate (BH) was sourced from the same tissue that was spiked with the plants for a comparison. An approximate one-log reduction in fluorescence signal can be seen from the extraction process; however, the addition of plant tissue homogenate prior to extraction does not seem to decrease the signal with respect to time to threshold (Figure 1). Time-to-threshold measurements for both the BH alone and the BH with plant tissue are not significantly different at more concentrated dilutions, which indicates that the method successfully removes the majority of interfering substances during prion extraction.

Dilutions out to 10^−4^ showed strong detection of PrP^TSE^ in *A. thaliana* and *B. distachyon* plants spiked with BH (Supplementary Figures S3 and S4). PrP^TSE^ with *Z. mays* was slightly more detectable in more dilute samples (Supplementary Figure S4). In all spiked plant experiments, the negative controls (plants combined with confirmed CWD-negative BH) did not show prion seeding activity for the duration of the assay.

### 3.3. Rinsing and Washing Experiments

**Figure 2:**
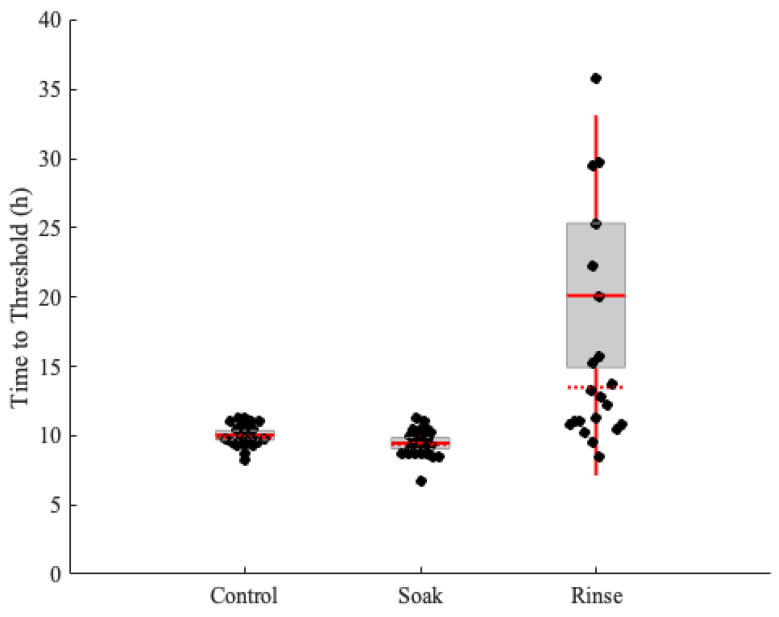
Prion deposition and removal from *Elymus repens* leaves. The box plots indicate the median time-to-threshold with a horizontal red line, the mean with a solid horizontal red line, the second and third quartiles with the box, and the first and fourth quartiles with the whiskers. *E. repens* leaves were exposed to CWD-positive brain homogenate and either soaked or rinsed with water, and then analyzed with real-time quacking-induced conversion (RT-QuIC).

To simulate a scenario where prions are deposited onto plant surfaces from animal excreta (urine, feces, saliva) and test whether plant tissue surfaces may retain prions, we tested if interactions with water can dislodge prions from leaf surfaces. Figure 2 shows the RT-QuIC time-to-threshold results of *E. repens* leaves when they are soaked in or rinsed with water.

There were no differences between the control leaves and the leaves that were soaked with water for 10 s after prion exposure. Rinsing with significant force does appear to decrease the concentration of seeding material; however, amyloid formation was still detectable in all replicates. These results are consistent with a previous study that demonstrated that plants exposed to scrapie prions and then rinsed remain infectious, as analyzed by both mouse bioassays and PMCA [27].

### 3.4. Assimilated Sample Experiments

**Figure 3:**
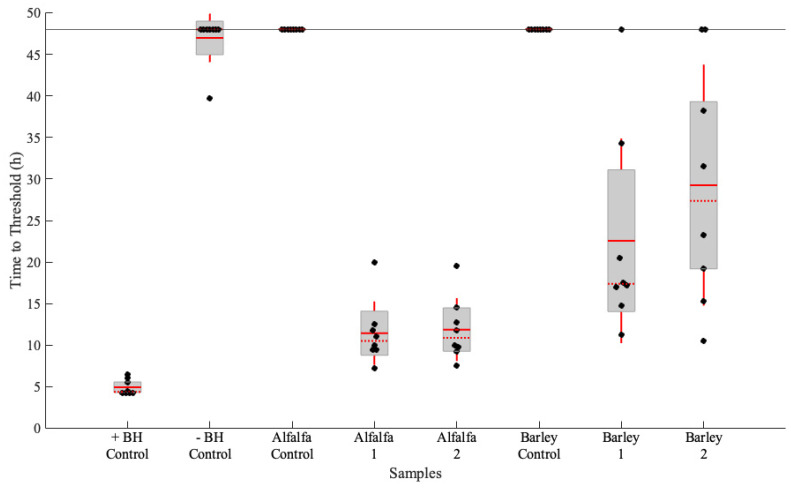
Confirmation of prion extraction method efficacy with plants that demonstrate prion seeding activity. Alfalfa and barley plants that were hydroponically grown in prion-spiked water were subjected to prion extraction and analyzed by real-time quaking-induced conversion (RT-QuIC). The box plots indicate the median time-to-threshold with a horizontal red line, the mean with a solid horizontal red line, the second and third quartiles with the box, and the first and fourth quartiles with the whiskers.

To confirm that the prion extraction method is indeed effective for prions that have been translocated into plants, samples from the previous study by Carlson et al. [28] were extracted and analyzed. These samples were stored at −80 °C for over ten years. The prion extraction method was performed on alfalfa and barley plants that had been hydroponically grown in mouse BH containing the RML strain of mouse-adapted scrapie (Figure 3). In addition, control experiments were conducted where commercially sourced alfalfa and barely were extracted, and the resultant extracts were analyzed by RT-QuIC alone (Figure 3) and with negative and positive BH spikes at a 10^−4^ dilution (Supplementary Figure S5). The alfalfa samples consisted of both leaves and stems, and the barley samples were only comprised of the leaves. All eight technical replicates in both alfalfa samples indicated amyloid formation, and their average time to thresholds were very similar. Despite the same treatment, barley samples showed slower amyloid formation than the alfalfa samples. Barley results still indicated prion seeding activity, though the time-to-threshold values were more varied than the alfalfa samples. Barley is a grass and therefore more fibrous than a legume such as alfalfa; for consistent results, grass samples may require more physical force to successfully homogenize.

In addition to confirmation of prion seeding activity, stems and leaves of alfalfa plants were separated and analyzed (Supplementary Figures S6 and S7). There were no statistical differences between the time-to-threshold of the stems and leaves of individual plants. A direct comparison between the Carlson et al. results and ours is not possible due to a lack of original sample material; however, our results in this study are consistent with the original findings, and samples were confirmed infectious by bioassay and PMCA, which allows for some level of qualitative comparison [28].

## 4. Discussion

Ultrasensitive protein amplification assays have rapidly become an indispensable tool for research into the biology and ecology of prion diseases. Though meaningful efforts have been made to correlate seeding activity and infectivity [2,13,50], there remain significant gaps in the literature related to the effects of context and environment on each. For instance, it is well established that the infectivity of prions can be altered by environmental deposition [51]. Likewise, it is plausible that seeding activity and infectivity may be influenced by plant uptake in ways that may not be immediately apparent or may be specific to the plant that has assimilated prions. Particularly relevant for this specific study, due to our protocol being extractive in nature, it is highly probable that we are underestimating the actual seeding material present in each of the samples analyzed. Future studies will be required to fully articulate the loss of seeding material by extraction and if those losses can be consistently accounted for in estimations of infectivity, with the latter point being especially salient when amplification assays are employed as a food safety measure.

This study is, to our knowledge, the first to demonstrate the efficacy of the RT-QuIC assay for detection of prion seeding activity within and on plant tissue surfaces. Practical and research applications of this method are numerous. For instance, routine plant sampling could be applied with antemortem animal testing in the context of captive cervids or pasture-fed ovines. In addition, plant sampling at potential CWD hotspot locations such as salt licks, carcass sites, and scrapes could be considered for future monitoring efforts. As much as ovine, bovine, and cervid diets consist of plant matter, even the consumption of plants with low concentrations of prions may be a significant TSE exposure and transmission pathway. Investigations into the hazard posed by prion-contaminated animal feeds are still comparatively rare and, due to the significant expense associated with live animal studies, comparatively risky. Using RT-QuIC, investigators will be able to confirm seeding activity in feeds prior to use in transmission studies, greatly reducing the risk of null results. Further research evaluating prion loads in different plant species grown in different soil types and under different environmental conditions may further our understanding of prion uptake by plants and the extent of relevant prion bioavailability, ultimately informing environmental surveillance and sampling efforts.

Another practical use of this technology is food safety and pathogen screening. The ability to detect prion seeding in plant tissues with high sensitivity will allow future research to address food safety concerns for produce, legumes, and grains grown in CWD-endemic areas. The transmission barrier of CWD prions to other mammals depends heavily on the route of transmission, and the risk of bovine, ovine, and human susceptibility is considered low but not insurmountable [52,53,54,55,56]. Should evidence of CWD or other zoonotic prion diseases transmitting to humans emerge, this technology will likely see widespread implementation in screening efforts.

It is important to note the long-term persistence of prion seeding activity in plants kept in storage for years. Some of the samples tested in this study were stored in a −80 °C freezer for a decade and still promoted prion seeding in RT-QuIC. Though this is not entirely surprising, given the incredible persistence of prions generally [57,58,59], there are important implications of these specific findings. Foremost among these is that pathogenic prion is likely to persist in foodstuffs, likely to a similar extent as seen in environmental samples. Further research looking at storage conditions (silo, open air storage, freeze dry, etc.) of legumes, grains, and other produce may find differences in prion bioavailability, which has been demonstrated in other prion-infected matrices such as tissues, feces, and soil [60,61,62].

## Data Availability

Data is contained within the article and Supplementary Material.

## Supplementary Materials

The following supporting information can be downloaded at: https://www.mdpi.com/article/10.3390/pathogens13060452/s1, Figure S1: The presence of plant material inhibits the real-time quaking induced conversion (RT-QuIC) assay; Figure S2: Prion extraction method experimental parameters analyzed with real-time quaking-induced conversion (RT-QuIC); Figure S3: Prion extraction and real-time quaking-induced conversion (RT-QuIC) analysis of chronic wasting disease (CWD) prion-spiked *Brachypodium distachyon* plant tissue; Figure S4: Prion extraction and real-time quaking-induced conversion (RT-QuIC) analysis of chronic wasting disease (CWD) prion-spiked maize and *Arabidopsis thaliana* plant tissues; Figure S5: Prion extraction and real-time quaking-induced conversion (RT-QuIC) analysis of chronic wasting disease (CWD) prion-spiked alfalfa and barley leaf tissue; Figure S6: Prion extraction and real-time quaking-induced conversion (RT-QuIC) analysis of barley leaf tissue hydroponically grown in prion contaminated water; Figure S7: Prion extraction and real-time quaking induced conversion (RT-QuIC) analysis of alfalfa stems and leaf tissue hydroponically grown in prion contaminated water.
