# Supplementary material for: Prion Seeding Activity in Plant Tissues Detected by RT-QuIC
Source: Pathogens. 2024 May 26;13(6):452. doi: 10.3390/pathogens13060452 (PMC11206635; doi:10.3390/pathogens13060452)
Supplement: Supplementary file 1 [file pathogens-13-00452-s001.zip › pathogens-2924893-supplementary.pdf]

Supplemental Information

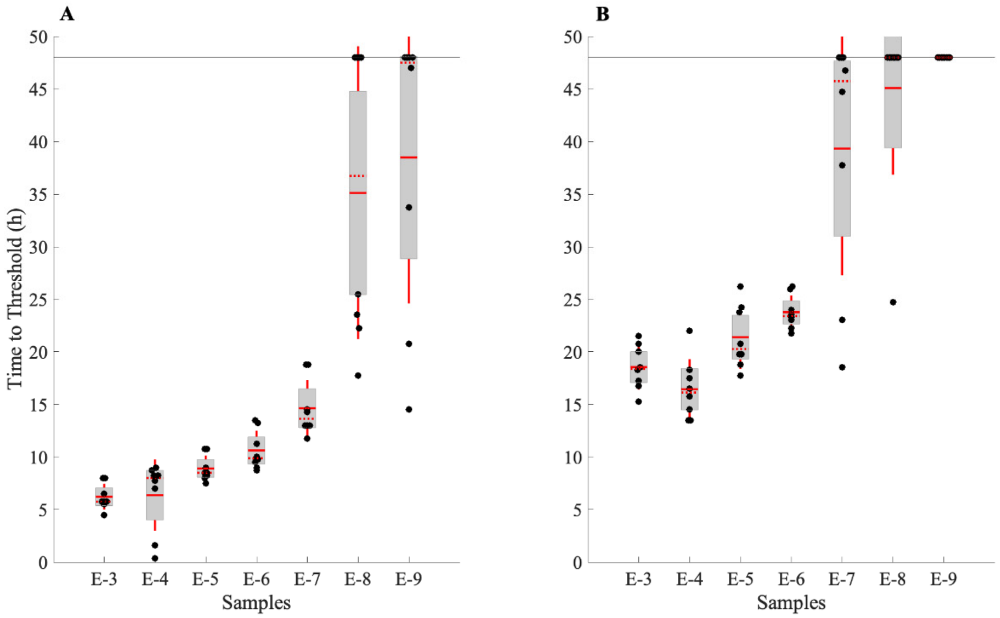

Supplemental Figure S1. The presence of plant material inhibits the real-time quaking induced conversion (RT-QuIC) assay. Box plots indicate the median time-to-threshold with a dashed horizontal red line, mean with a solid horizontal red line, second and third quartiles with the box, and first and fourth quartiles with the whiskers. *Brachypodium distachyon* leaves were homogenized and spiked with the indicated dilution of CWD+ brain homogenate and analyzed by RT-QuIC. A – Unspiked brain homogenate B – *B. distachyon* spiked with brain homogenate. Note that the brain homogenate used in this experiment differs from the brain homogenate used in all other experiments in this study, and a different batch of truncated recombinant hamster prion protein was used as well.

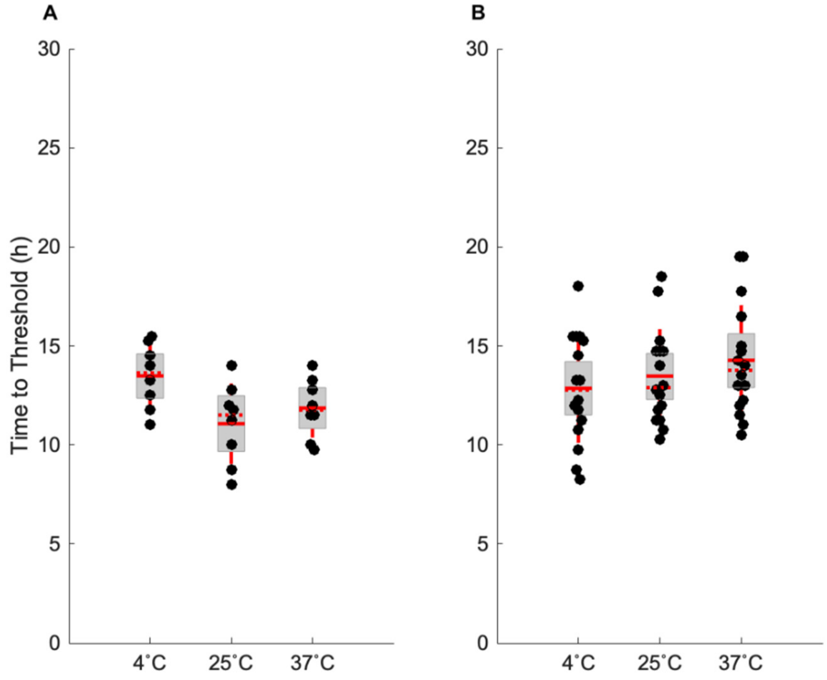

Supplemental Figure S2. Prion extraction method experimental parameters analyzed with real-time quaking-induced conversion (RT-QuIC). Box plots indicate the median time-to-threshold with a dashed horizontal red line, mean with a solid horizontal red line, second and third quartiles with the box, and first and fourth quartiles with the whiskers. Leaves from *Elymus repens* plants were spiked with brain homogenate (BH) and underwent prion extraction. The temperature and duration of the sodium phosphotungstate (NaPTA) precipitation step was varied to see if differences in the protocol influenced extraction results. (A) NaPTA precipitation of 1 hour at three different temperatures. (B) 16-hour NaPTA precipitation at three different temperatures.

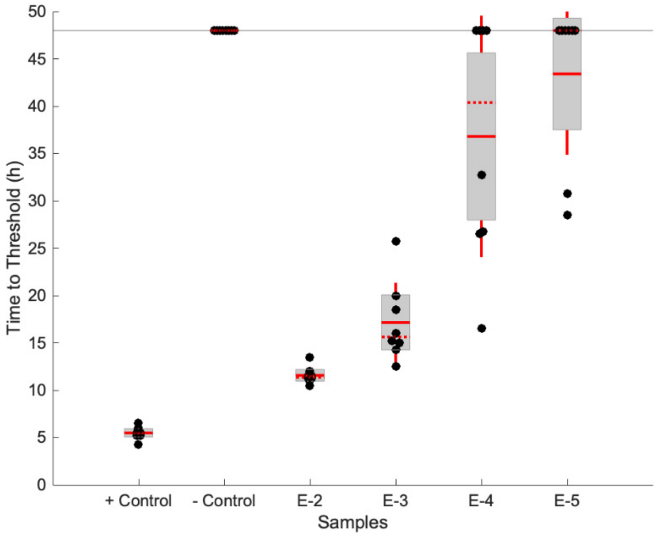

Supplemental Figure S3. Prion extraction and real-time quaking-induced conversion (RT-QuIC) analysis of chronic wasting disease (CWD) prion-spiked *Brachypodium distachyon* plant tissue. Box plots indicate the median time-to-threshold with a horizontal dashed red line, mean with a horizontal solid red line, second and third quartiles with the box, and first and fourth quartiles with the whiskers. The horizontal black line at 48 h indicates the end time of the assay. The positive control is known CWD-positive obex brain homogenate (BH) at 10^{-3} dilutions. The negative control is *B. distachyon* tissue spiked with known CWD-negative obex BH which was subjected to the prion extraction method. *B. distachyon* was spiked with a series of CWD-positive BH dilutions and then subjected to the prion extraction method. As BH is serially diluted, the time to threshold and variation between replicates increases.

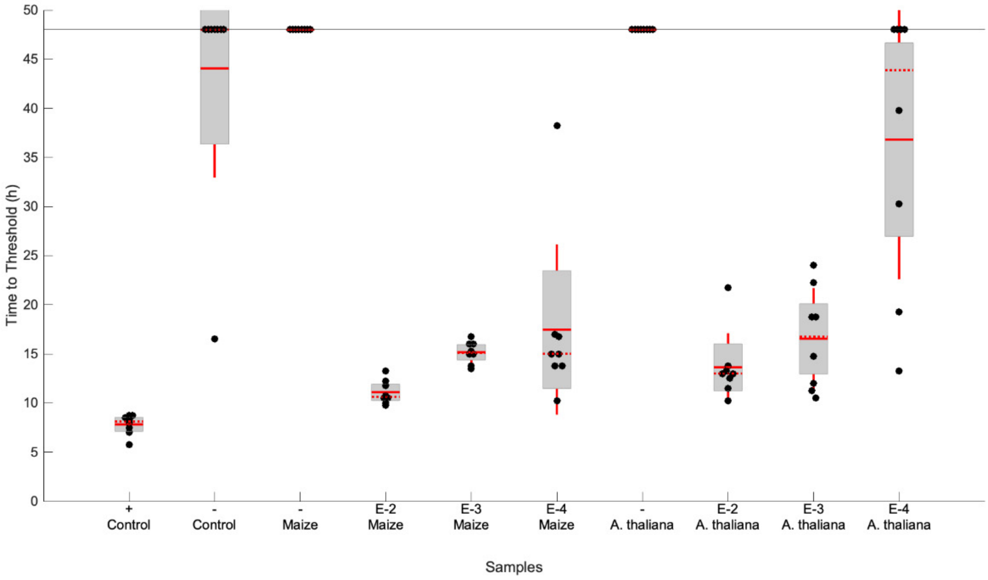

Supplemental Figure S4. Prion extraction and real-time quaking-induced conversion (RT-QuIC) analysis of chronic wasting disease (CWD) prion-spiked maize and *Arabidopsis thaliana* plant tissues. Box plots indicate the median time-to-threshold with a horizontal dashed red line, mean with a horizontal solid red line, second and third quartiles with the box, and first and fourth quartiles with the whiskers. The horizontal black line at 48 h indicates the end time of the assay. The positive and negative controls are known CWD-positive and negative obex brain homogenates (BH) at 10^{-3} dilutions. The plant controls (i.e. '- Maize' and '- *A. thaliana*') are plant tissues spiked with CWD-negative BH that were extracted and analyzed. Both CWD-positive spiked maize and *A. thaliana* were extracted and dilutions of 10^{-2} to 10^{-4} were analyzed for amyloid formation with RT-QuIC.

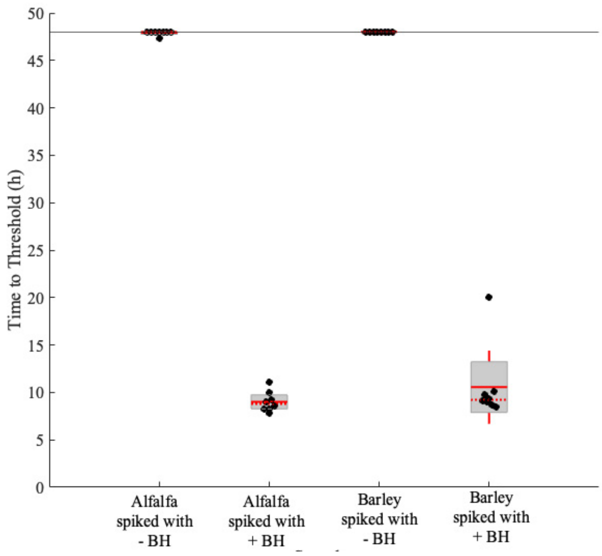

Supplemental Figure S5. Prion extraction and real-time quaking-induced conversion (RT-QuIC) analysis of chronic wasting disease (CWD) prion-spiked alfalfa and barley leaf tissue. Box plots indicate the median time-to-threshold with a horizontal dashed red line, mean with a horizontal solid red line, second and third quartiles with the box, and first and fourth quartiles with the whiskers. Alfalfa and barley were commercially sourced and spiked with both CWD-positive and CWD-negative obex brain homogenate (BH) at 10^{-4} dilutions. The horizontal black line at 48 h indicates the end time of the assay.

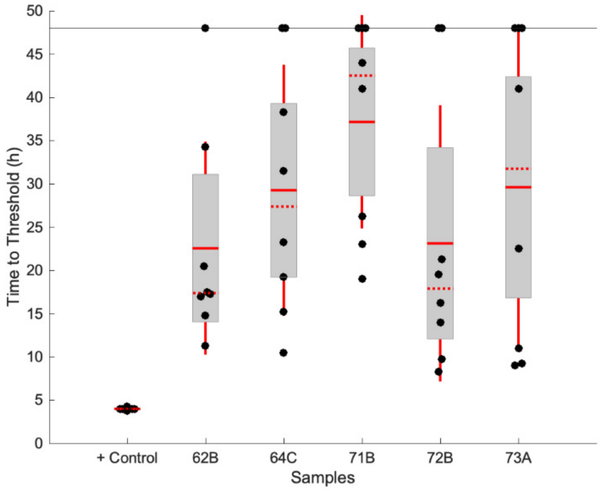

Supplemental Figure S6. Prion extraction and real-time quaking-induced conversion (RT-QuIC) analysis of barley leaf tissue hydroponically grown in prion contaminated water. Box plots indicate the median time-to-threshold with a horizontal dashed red line, mean with a horizontal solid red line, second and third quartiles with the box, and first and fourth quartiles with the whiskers. The positive control is known chronic wasting disease (CWD)-positive obex brain homogenate at 10^{-3} dilutions. The horizontal black line at 48 h indicates the end time of the assay. Five barley plants were subjected to the extraction method protocol and amyloid formation was analyzed with RT-QuIC.

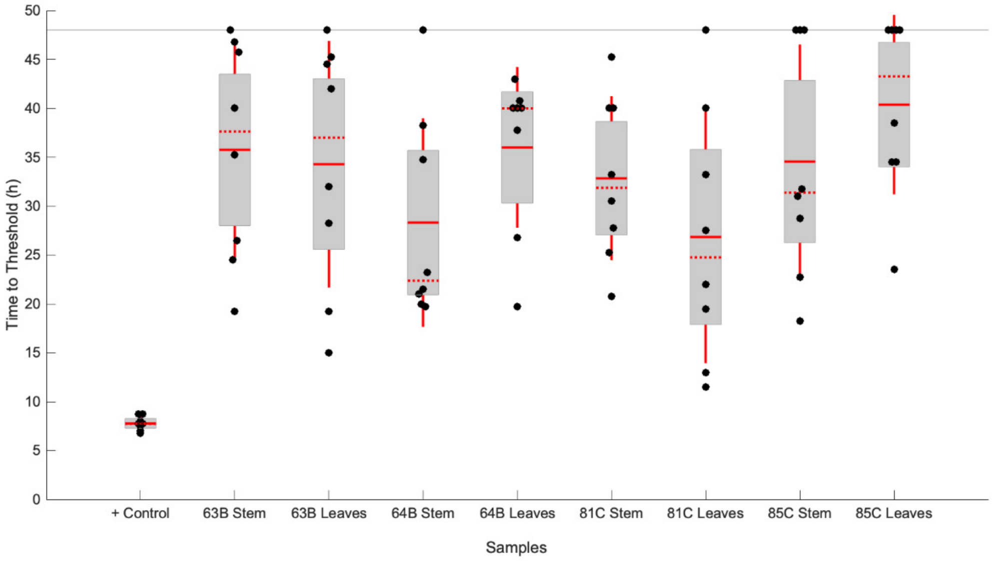

Supplemental Figure S7. Prion extraction and real-time quaking induced conversion (RT-QuIC) analysis of alfalfa stems and leaf tissue hydroponically grown in prion contaminated water. Box plots indicate the median time-to-threshold with a horizontal dashed red line, mean with a horizontal solid red line, second and third quartiles with the box, and first and fourth quartiles with the whiskers. The positive control is known chronic wasting disease (CWD)-positive obex brain homogenate at 10^{-3} dilutions. The horizontal black line at 48 h indicates the end time of the assay. Four alfalfa plants' stems and leaves were subjected to the extraction method protocol and amyloid formation was analyzed with RT-QuIC. None of the leaf/stem pairings were significantly different with a double-sided t-test.
